# INNOVATING LONG-TERM CARE DURING COVID-19 ACROSS FOUR NATIONS: INNOVATIONS, BARRIERS, AND FACILITATORS

**DOI:** 10.1093/geroni/igae098.2159

**Published:** 2024-12-31

**Authors:** Charlene Chu, Ruth Melo, Barbara Bowers

**Affiliations:** Chair; Co-Chair; Discussant

## Abstract

The COVID-19 pandemic has starkly highlighted the vulnerabilities of older adults in residential long-Term Care Homes (LTCHs), amplifying calls to “reimagine” the LTC sector. Active engagement and inclusion of older adults, their families, and staff in the development and implementation of LTC innovations have been recommended by the United Nations and the World Health Organization, but little is known about such engagement in innovations. This symposium presents a comprehensive scoping review aimed at understanding innovations implemented in LTCHs in four countries (Brazil, Canada, Switzerland, United States) since 2020, and the practices of inclusion within innovation processes within LTCHs. The first paper outlines the scoping review methodology and summarizes national-level results, including the number of studies per country and types of studies (e.g., observational, experimental). The second paper presents an analysis of interventions deployed across different countries. The third paper focuses on the types and goals of technology-based innovations implemented in LTCHs during COVID-19 and summarizes the extent to which perspectives of residents, staff, and family caregivers were considered regarding technology-based innovations. The final paper examines the barriers and facilitators of the innovation process during COVID-19. This international symposium contributes to the reimagining and transformation of LTCHs into more resilient, inclusive, and high-quality living environments.

